# Sedentary behaviour among elderly patients after total knee arthroplasty and its influencing factors

**DOI:** 10.1038/s41598-024-64836-5

**Published:** 2024-06-20

**Authors:** Xuan Ni, Juan Shi, Qing Hu, Ai Li, Xing Zeng, Yajing Gu

**Affiliations:** 1 Department of Orthopedics, The Affiliated Jiangning Hospital with Nanjing Medical University, Nanjing, Jiangsu 211100 China; 2 Nursing Department, The Affiliated Jiangning Hospital with Nanjing Medical University, Nanjing, Jiangsu China; 3 Department of Gastrointestinal Oncology Surgery, The Affiliated Jiangning Hospital with Nanjing Medical University, Nanjing, Jiangsu China; 4 Department of Urology, The Affiliated Jiangning Hospital with Nanjing Medical University, Nanjing, Jiangsu China

**Keywords:** Knee arthroplasty, Sedentary behaviour, Socio-ecological model, Influencing factors, Psychology, Ecology, Diseases

## Abstract

To understand the status of sedentary behaviour in elderly patients after total knee arthroplasty and analyse its influencing factors so as to provide a reference for developing targeted interventions. Conveniently selected elderly patients undergoing total knee arthroplasty (> 6 months) in a tertiary hospital in Jiangsu Province were investigated using a general information questionnaire, the Charlson Comorbidity Index, patients’ self-reported sedentary behaviour information, the WOMAC Score, The Groningen Orthopaedic Social Support Scale, and Lee’s Fatigue. The median daily sedentary time was 5.5 h (4.5 h, 6.625 h) in 166 elderly patients after total knee arthroplasty, of whom 82 (49.40%) showed sedentary behaviour (≥ 6 h per day). Logistic regression analysis showed that being retired/unemployed (OR = 8.550, 95% CI 1.732–42.207, *P* = 0.0084), having a CCI score ≥ 3 (OR = 9.018, 95% CI 1.288–63.119, *P* < 0.0001), having high WOMAC scores (OR = 1.783, 95% CI 1.419–2.238, *P* < 0.0001), having a high social support score (OR = 1.155, 95% CI 1.031–1.294, *P* = 0.0130), and having a fatigue score ≥ 5 (OR = 4.848, 95% CI 1.084–21.682, *P* = 0.0389) made patients more likely to be sedentary. The sedentary time of elderly patients after total knee arthroplasty is long, and sedentary behaviour is common among them. Healthcare professionals should develop targeted sedentary behaviour interventions based on the influencing factors of sedentary behaviour in order to reduce the occurrence of sedentary behaviour in elderly patients after total knee arthroplasty.

## Introduction

Population ageing has become one of China’s key concerns. According to the results of the seventh national census, the total number of persons over 60 years of age in mainland China is 264 million, accounting for 18.7% of the country’s total population. The proportion of this age group has increased by 5.44% compared with the results of the sixth national census conducted ten years ago^1^. This major demographic change poses serious challenges to the economy and the healthcare system because age-related conditions, such as osteoarthritis, impose considerable financial and psychological burdens on individuals, families, and society^2^. Osteoarthritis (OA) is a degenerative disease with joint pain as the main symptom, which is caused by fibrosis, ulceration, and loss of articular cartilage due to a variety of factors. Osteoarthritis of the knee (KOA) is the most common type, and the prevalence of symptomatic KOA is reported to be 10.3% (women) and 5.7% (men) in older Chinese individuals^3^. Total knee arthroplasty (TKA) is considered to be the most effective treatment for end-stage KOA, and the procedure typically results in substantial reductions in pain and improvements in physical function^4^.

Evidence shows that older adults (aged ≥ 60 years) are the most sedentary segment of the population^5^. Specifically, they spend approximately 80% of their waking hours (8–12 h per day) with sedentary activities^6^. Sedentary behaviours, also known as static behaviours, are defined as any waking energy expenditure < 1.5 metabolic equivalents (METs), including sedentary activities such as sitting, reclining, or lying down, use of electronic devices, writing, reading, talking, and riding on public transport^7^. As for elderly TKA patients, sedentary behaviour (SB), which is associated with an increased risk of multiple adverse health outcomes, has become a concern for clinical experts and researchers, but few relevant studies on this topic have been conducted in China. Some quantitative studies abroad have focused on the comparison between SB and physical activity (PA) in preoperative and postoperative TKA patients^4,8^. A few studies used accelerometers to objectively measure SB in post-TKA patients, and the results showed that numerous factors affect SB in post-TKA patients, mainly age^9^, body mass index (BMI)^10^, gender^9^, occupation^11^, education level^12^, already having a sedentary lifestyle^10,13^, and one’s psychological state^13^ before the operation, but the factors influencing of sedentary behaviours in elderly postoperative TKA patients remain unclear. In addition, most of the existing studies on the influencing factors of SB lack a theoretical framework, and the analysis results are scattered. The health ecological model (HEM) states that individual health behaviours are the result of a combination of individual and environmental factors^14^, a theory that fits well with the fact that SB is influenced by multidimensional factors.

Therefore, based on the HEM, this paper investigates the current status of SB in elderly Chinese post-TKA patients, further analyses the influencing factors of SB to complement and enrich the results of previous quantitative studies, and provides a theoretical basis for the development of more systematic and targeted interventions to reduce SB in elderly post-TKA patients.

## Materials and methods

### Study design, study site, and population characteristics

A cross-sectional study design was employed. The study was carried out in the Department of Orthopaedics of a tertiary care hospital in Jiangsu Province between June 2022 and June 2023. All patients included in this study were diagnosed with KOA, underwent TKA done by the same surgeon, and freely agreed to participate in the study.

#### Inclusion criteria

① Speaking Chinese and being aged older than 60 years; ② Meeting the diagnostic criteria for KOA as described in the Chinese guidelines for the diagnosis and treatment of osteoarthritis (2021 edition), with a Kellgren Lawrence classification of III–IV; ③ Having undergone unilateral TKA under general anaesthesia during hospitalisation (initial TKA on the operated side) > 6 months ago; ④ Possessing normal abilities to understand information and communicate; ⑤ Knowing the details of the study and signing the informed consent form.

#### Exclusion criteria

① Currently undergoing bilateral knee replacement or a postoperative revision for TKA; ② Having malignant tumours or severe organ dysfunction; ③ Being unable to stand without assistance after surgery (assistive devices, such as canes or walkers, are permitted); ④ Lost to follow-up.

### Sampling method and technique

A convenience sampling technique was used to select participants who met the inclusion criteria. The sample size for this cross-sectional study was calculated using the Kendal^15^ sample size estimation formula: N = number of total variables*(5 ~ 10) times the total number of independent variables investigated, which is 15 in this study. According to the formula, the necessary sample size was calculated to be 75 ~ 150 cases, considering a 20% missed-visit rate, so the required sample size was considered to be 90 ~ 180 cases. A total of 175 questionnaires were distributed in this study, and 166 valid questionnaires were recovered, with an effective response rate of 94.86%.

### Variables

After the literature review, the representative factors of each dimension of the HEM were initially organised. Using the focus group discussion method, influential factors that may affect sedentary behaviour among patients after TKA were included after two rounds of subject group validation in order to systematically explore the current situation and the factors influencing sedentary behaviour in elderly postoperative TKA patients. These factors included ① Personal characteristics: age, gender, BMI, educational level, and comorbidities; ② Psychological and behavioural characteristics: Preoperative sedentary behaviour, understanding of the hazards of prolonged sitting, fatigue, and knee function; ③ Interpersonal network: marital status and social support; ④ Living and working conditions: Residential status, occupational status, and monthly income; ⑤ Policy environment: type of health insurance.

### Instruments

#### Self-designed questionnaire for sociodemographic and clinical characteristics

We obtained patients’ general demographic data from the electronic medical record system, including their gender, age, comorbidities, education level, monthly income, and type of medical insurance. Patients self-reported their marital status, occupational status, residential status, whether they had a sedentary lifestyle before surgery, whether they were aware of the dangers of sitting, and their height and weight for the calculation of their body mass index (BMI). According to the recommended BMI cut-off values < 24 kg/m^2^, 24.0–27.9 kg/m^2^, and ≥ 28.0 kg/m^2^ for Chinese adults, patients were classified as underweight or normal weight, overweight, or obese, respectively^16^.

#### Charlson comorbidity index (CCI)

The Charlson Comorbidity Index score was created by Charlson^17^. It consists of 19 diseases and uses the patient’s underlying disease as the index for scoring, assigning different scores of 1, 2, 3, or 6 to each index, which are then added to create a total score of 0–36: The score is increased by 1 point for every 10 years of age over 40 years, with higher scores being associated with greater severity of comorbidities. Based on the questionnaire scores, the severity of comorbidities can be classified into three categories: mild (< 3 points), moderately severe (≥ 3 points)^17^.

The CCI has shown sound psychometric properties, including predictive, concurrent, and incremental validity. In addition, it has shown good inter-rater reliability, which was confirmed in the current study with an inter-rater reliability of 0.96.

#### Western ontario and mcmaster universities osteoarthritis index (WOMAC)

WOMAC was used to assess knee function in elderly post-TKA patients. The WOMAC was developed by Canadian scholars Bellamy^18^ in 1988 and adapted for the Chinese context by Xie et al.^19^. It consists of the three dimensions of pain, stiffness, and difficulty with performing daily activities, with a total of 24 entries, and is rated on a 5-point Likert scale of 0 (no pain or difficulty) to 4 (extreme pain or difficulty), with higher scores indicating poorer knee function. The Cronbach’s α for the three dimensions of the Chinese version of the WOMAC scale were 0.82, 0.88, and 0.84, respectively^19^. The current WOMAC is one of the most commonly used patient-reported outcome measures.

#### Patient self-reported sedentary behaviour

The method of assessing sedentary behaviour used in Teychenne et al.’s^20^ study was used to assess sedentary behaviour in this study, in which the study participants self-report their daily time spent sitting, including on transport, commuting to work, watching TV, using a computer/mobile phone, and taking other breaks (reading newspapers/books, playing cards, playing chess) during weekdays, as well as their daily time spent sitting during any of the abovementioned activities during the weekends, which was calculated by using the formula: daily sedentary time = (weekday sitting time × 5 + weekend sitting time × 2)/7. In this study, we referred to previous relevant literature^13,21^ and defined behaviour as sedentary when the total sedentary time was ≥ 6 h/d and as non-sedentary when the total sedentary time was < 6 h/d.

#### Groningen orthopaedic social support scale (GO-SSS)

In 2004, Dutch scholars Akker-Scheek et al. developed the Groningen Orthopaedic Social Support Scale (GO-SSS) to assess the social support postoperative orthopaedic patients receive, and the Cronbach’s α coefficient of the scale was 0.89^22^. Adapted to the Chinese context by Sheng et al.^23^ the scale consists of 12 questions divided into two subscales: perceived social support (7 items) and instrumental support (5 items), both of which are rated on a four-point Likert scale, with answer categories ranging from “never” to “often”, with a total score of 0–3, in which a higher the score indicates better social support. The GO-SSS proved to be a reliable and valid instrument to assess social support for patients following arthroplasty, with a 0.863 Cronbach’s α for the entire questionnaire^24^.

#### Lee fatigue scale (LFS)

The Chinese version of the Lee Fatigue Scale (LFS) was used to measure fatigue severity^25^. Fatigue severity was rated by seven items in the Chinese version of the LFS, rating each item on a numeric rating scale ranging from 1 to 10. A total score was calculated in the form of the mean of the seven items, with higher scores indicating greater fatigue severity. For this study, scores ≥ 5 were considered indicative of severe fatigue. The LFS has adequate psychometric properties^26^ and is able to reduce the burden on the respondent. It is suitable for older adults and was chosen in consideration of the characteristics of the population included in this study.

### Data collection

The data collection method was jointly completed by two postgraduate students with unified training. Ten discharged post-TKA patients were selected for a pre-survey to assess whether the scale or questionnaire was easy for them to understand and to adjust any ambiguous or difficult statements. The pre-survey revealed that a significant proportion of the patient group were farmers, who were considered to be in a working condition in this study, and their level of physical activity was judged based on enquiries about their daily labour time and their degree of knee activity, and they were categorised as unemployed if their labour time and intensity were low. Patients were informed of the purpose and significance of the study when they were discharged from the hospital, promised that their information would be kept confidential and used only for this study, and all participants signed an informed consent form. Participants were surveyed and data were collected 6 months after surgery via telephone or WeChat (a social media and messaging application widely used in China) communication.

### Ethical considerations

Guided by the 2000 Declaration of Helsinki for ethical standards, the protocol was approved by the Committee on the Ethics of Medical Research of the investigating hospital (2023-03-035-K01). Informed consent was provided by all participants prior to their participation. The survey was anonymous, and the confidentiality of the information was assured.

### Validity and reliability/rigor

The study was conducted and reported under the Strengthening the Reporting of Observational Studies in Epidemiology (STROBE) guidelines for cross-sectional studies.

### Statistical analysis

The collected data were entered and analysed using SAS 9.4. The Shapiro–Wilk method was used to test the normality. The measurement data of the normal distribution were represented by mean ± standard deviation ($\overline{x} \pm S$), and the comparison between the two groups was performed by an independent sample t test. The measurement data of the non-normal distribution were represented by M (P25, P75), and the rank sum test of two independent samples (Mann-Whittney U test) was used to compare the two groups. Descriptive analyses were performed using frequencies and percentages, and the χ^2^ test was used to compare statistical data between groups. The statistically significant indicators after the single-factor analysis were incorporated into the multi-factor logistic regression model to identify the risk factors of sitting for too long. A *p* value < 0.05 was considered statistically significant.

## Results

### Participant characteristics

The results of the study showed that the daily ST of elderly post-TKA patients ranged from 2.5 to 10.5 h/d, with a median of 5.5 h (P25, P75:4.5 h, 6.625 h), and 49.40% of participants were sedentary for ≥ 6 h/d. Further stratified by ST, 84 patients had an ST < 6 h/d, accounting for 50.6% of participants; 70 patients had an ST of 6–8 h/d, accounting for 42.2% of participants; 12 patients had an ST ≥ 8 h/d, accounting for 7.2% of participants.

### Univariate analysis of sedentary behaviour in elderly post-TKA patients

Of the 166 elderly post-TKA patients included in the study, 36 were male (21.69%) and 130 were female (78.31%); their ages ranged from 60 to 82 (68.93 ± 5.07) years, of which 59 (35.54%) were ≥ 70 years old. 24.70% of participants are underweight/normal(BMI < 24), 47.59% of participants are overweight (BMI 24.0–27.9), and 27.71% of participants are obese(BMI ≥ 28.0). In terms of marital status, 133 (80.12%) were married. Most participants lived with their spouse (48.8%) or with their children (38.55%). The education status was primary school and below for 98 (59.04%), secondary school for 49 (29.52%), and tertiary school and above for 19 (11.44%). According to Jiangsu Province 2023 Statistical Yearbook: per capita disposable income in Jiangning District is about 6400 RMB/month. The monthly income was < 4000 RMB for 109 (65.66%), 4000 ~ 6000 RMB for 33 (19.88%), and ≥ 6000 RMB for 24 (14.46%). The patient characteristics showing statistically significant differences in terms of sedentary behaviour among patients are shown in Table 1.

**Table 1:** The patient characteristics showing differences in terms of SB among patients.

Variables	Number	Non-sedentary	Sedentary	χ^2^/t/z	*P*-value
		(n = 84)	(n = 82)		
Age [n(%)]				10.2175	0.0014
< 70	107	64(59.8)	43(40.2)		
≥ 70	59	20(33.9)	39(66.1)		
BMI [kg/m^2^, n(%)]				8.5562	0.0139
< 24.0	41	28(68.3)	13(31.7)		
24.0–27.9	79	39(49.4)	40(50.6)		
≥ 28.0	46	17(37.0)	29(63.0)		
Occupational status [n(%)]				25.2307	< 0.0001
Working/farming	73	53(72.6)	20(27.4)		
Retired/unemployed	93	31(33.3)	62(66.7)		
Residential status [n(%)]				6.3541	0.0417
Living with spouse	81	43(53.1)	38(46.9)		
Living with children	64	26(40.6)	38(59.4)		
lived alone/in a nursing home/other	21	15(71.4)	6(28.6)		
CCI [n(%)]				14.5432	0.0001
< 3	49	36(73.5)	13(26.5)		
≥ 3	117	48(41.0)	69(59.0)		
WOMAC [IQR]	166	18(16–21)	28(26–31)	10.0204	< 0.0001
Understanding of the hazards of prolonged sitting [n(%)]				18.2202	< 0.0001
No	96	35(36.5)	61(63.5)		
Yes	70	49(70.0)	21(30.0)		
Preoperative sedentary behaviour [n(%)]				5.4434	0.0196
Yes	96	56(58.3)	40(41.7)		
No	70	28(40.0)	42(60.0)		
GO‐SSS [Mean ± SD]	166	20.82 ± 5.43	24.17 ± 6.20	− 3.71	0.0003
Fatigue [n(%)]				18.0589	< 0.0001
< 5	72	50(69.4)	22(30.6)		
≥ 5	94	34(36.2)	60(63.8)		
Gender [n(%)]				1.0991	0.2945
Male	36	21(58.3)	15(41.7)		
Female	130	63(48.5)	67(51.5)		
Educational level [n(%)]				3.1892	0.2030
Primary school and below	96	43(44.8)	53(55.2)		
Secondary school	49	28(57.1)	21(42.9)		
Tertiary school and above	21	13(61.9)	8(38.1)		
Monthly income [n(%)]				4.1123	0.1279
< 4000 RMB	109	55(50.5)	54(49.5)		
4000–6000 RMB	33	13(39.4)	20(60.6)		
≥ 6000 RMB	24	16(66.7)	8(33.3)		
Type of health insurance [n(%)]				1.2045	0.5476
Urban health Insurance	58	26(44.8)	32(55.2)		
Rural health insurance	77	41(53.3)	36(46.8)		
No health insurance	31	17(54.8)	14(45.2)		
Marital status [n(%)]				0.8013	0.3707
Married	133	65(48.9)	68(51.1)		
Single (divorced/widowed/unmarried)	33	19(57.6)	14(42.4)		

*BMI* Body mass index; *CCI* The Charlson Comorbidity Index; *Failure* Lee’s Fatigue Scale; *GO-SSS* The Groningen Orthopaedic Social Support Scale; *WOMAC* The Western Ontario and McMaster Universities Osteoarthritis Index.

### Logistic regression analysis of sedentary behaviour in elderly post-TKA patients

Ten variables, including age, BMI, occupational status, residential status, CCI score, WOMAC score, whether the patient understands the harms of sedentary behaviour, whether they exercise regularly, the social support score, and the fatigue score, were included in the multifactorial logistic regression analyses (see Table 2 for the assignment of the variables). The results of the logistic regression model showed that the five variables, namely, occupational status, CCI score, WOMAC score, social support score, and fatigue score, were all risk factors for sedentary behaviour.

**Table 2:** The assignment of the variables.

Variables	Assignment situation
Age	< 70 = 1, ≥ 70 = 2
BMI	< 24.0 = 1, 24.0–27.9 = 2, ≥ 28.0 = 3
CCI	< 3分 = 1, ≥ 3分 = 2
Occupational status	Working/farming = 1, retired/unemployed = 2
Residential status	Living with spouse = 1, living with children = 2, living alone/nursing home/other = 3
Understanding of the hazards of prolonged sitting	No = 1, Yes = 2
Preoperative sedentary behaviour [n(%)]	No = 1, Yes = 2
WOMAC	Original value input
GO‐SSS	Original value input
Fatigue	< 5 = 1, ≥ 5 = 2

*BMI* Body mass index; *CCI* Charlson Comorbidity Index; *Failure* Lee’s Fatigue Scale; *GO-SSS* The Groningen Orthopaedic Social Support Scale; *OR* Odds Ratio; *SE* Standard Error; *WOMAC* The Western Ontario and McMaster Universities Osteoarthritis Index.

Those who were retired/unemployed (OR = 8.550, 95% CI 1.732–42.207, *P* = 0.0084), had a CCI score ≥ 3 (OR = 9.018, 95% CI 1.288–63.119, *P* < 0.0001), a high WOMAC score (OR = 1.783, 95% CI 1.419–2.238, *P* < 0.0001), a high social support score (OR = 1.155, 95% CI 1.031–1.294, *P* = 0.0130), and a fatigue score ≥ 5 (OR = 4.848, 95% CI 1.084–21.682, *P* = 0.0389) were more likely to be sedentary (see Table 3).

**Table 3:** Adjusted odds ratios (OR) for SB.

Variable	Β	SE	Wald χ^2^	OR	95% CI	*P*-value
Intercept	− 21.5930	4.3493	24.6489			< 0.0001
Age						
< 70				1.00		
≥ 70	1.5790	0.9495	2.7655	4.850	(0.754–31.190)	0.0963
BMI (kg/m^2^)						
< 24.0				1.00		
24.0–27.9	0.6638	0.9001	0.5438	1.942	(0.333–11.335)	0.4608
≥ 28.0	1.6947	1.0203	2.7591	5.445	(0.737–40.224)	0.0967
Occupational status						
Working/farming				1.00		
Retired/unemployed	2.1459	0.8146	6.9387	8.550	(1.732–42.207)	0.0084
Residential status						
Living with spouse				1.00		
Living with children	0.4288	0.7447	0.3315	1.535	(0.357–6.609)	0.5648
Living alone/nursing home/other	0.2077	1.3429	0.0239	1.231	(0.089–17.110)	0.8771
CCI						
< 3				1.00		
≥ 3	2.1992	0.9928	4.9073	9.018	(1.288–63.119)	0.0267
WOMAC	0.5780	0.1162	24.7489	1.783	(1.419–2.238)	< 0.0001
Understanding of the hazards of prolonged sitting						
No				1.00		
Yes	− 0.5241	0.7000	0.5607	0.592	(0.150–2.334)	0.4540
Preoperative sedentary behaviour [n(%)]						
No				1.00		
Yes	0.4244	0.8193	0.2683	1.529	(0.307–7.616)	0.6045
GO‐SSS	0.1440	0.0580	6.1664	1.155	(1.031–1.294)	0.0130
Fatigue						
< 5				1.00		
≥ 5	1.5787	0.7642	4.2673	4.848	(1.084–21.682)	0.0389

*CI* Confidence Interval; *CCI* Charlson Comorbidity Index; *Failure* Lee’s Fatigue Scale; *GO-SSS* The Groningen Orthopaedic Social Support Scale; *OR* Odds Ratio; *SE* Standard Error; *WOMAC* The Western Ontario and McMaster Universities Osteoarthritis Index.

## Discussion

### SB is common in elderly post-TKA patients

In this study, 166 elderly TKA patients were investigated to differentiate between sedentary and non-sedentary individuals on the basis of ≥ 6 h of sedentary time per day. The results of this study showed that the daily SB incidence in elderly TKA patients was 49.40%. The median ST in TKA patients was 5.5 h (4.5 h, 6.625 h), which was lower than the results of the previous studies^4,27,28^. This difference can be attributed to multiple factors. On the one hand, SB surveys of rural residents in Henan^29^ and Zhejiang^30^, China, found that men sat for longer periods of time than women, which was attributed to the traditional life pattern in China, in which women had to take on greater responsibility for household chores and had fewer opportunities to be sedentary. Further, due to the characteristics of the population with KOA, the proportion of female patients in this study was high (78.31%). On the other hand, the subjects of this study were elderly patients with moderate-to-severe KOA who were treated with TKA. This means that the patients had already suffered from joint pain and immobility for a long period of time before the operation, and their increased joint mobility after the operation might lead to more participation in social activities and shorter ST. Meanwhile, this study used self-reports to collect patients’ SBs, and previous studies reported a gap between objective and subjective assessment tools as older adults are usually reluctant to identify themselves as sedentary, preferring to emphasise their physical activity and denying that they spend an excessive amount of time sitting^31^. In conclusion, the findings suggest that the current situation of SB in older TKA patients with longer ST is not optimistic and requires significant attention from clinical healthcare workers, as well as effective intervention strategies in order to prevent or mitigate the adverse health outcomes caused by SB. In addition, despite our pre-discharge patient education, it was found that about 57.83% (96/166) of the patients still did not understand the dangers of sedentariness.

According to the knowledge‐attitude‐practice theory, only when a patient’s knowledge of the disease has reached a certain level can it trigger a change in attitude^32^. This suggests that social media can be used to publicise and popularise knowledge related to SB reduction, and the community can regularly organise health lectures to share knowledge related to sedentary activities and repeatedly explain it in easy-to-understand language with habit formation as the focus of intervention to improve patients’ awareness of the consequences of prolonged sedentary activities and achieve the transformation from a mere intention into maintained behaviours.

### Occupational status, comorbidities, knee function, social support, and fatigue have an impact on SB in elderly post-TKA patients

Occupational status is an influential factor in sedentary behaviour among elderly TKA patients, and retired/unemployed elderly TKA patients were more inclined to be sedentary, using working/farming patients as a reference group. In China, the retirement age is 55 years old for women and 60 years old for men. After retirement, the elderly may seek employment because of economic pressure or because they cannot afford to simply stay at home, while employed/farming patients have a fixed daily physical labour time, leading to less sedentary time. Retired/unemployed patients had more leisure time at home and were, thus, prone to prolonged sedentary time. Hylkema et al.^33^ reported on 51 patients who returned to work one year after TKA and found that SB occurred more during leisure time (65.2%) than during working time (55.8%). This suggests that community geriatric care workers should pay more attention to SB in the home-bound elderly group, and instruct retired/unemployed patients to use their leisure time to develop more dynamic hobbies, such as gardening, tai chi, ba duan jin, etc., in order to increase their time spent with physical activity and reduce their sedentary time.

The results of the present study showed that sedentary behaviour in elderly post-TKA patients was influenced by comorbidities, similar to the findings of Da Silva et al.^34^ and Dörenkamp et al.^35^. Comorbidities are common in the elderly, but the exact mechanism of causality between comorbidities and sedentary behaviour has not been fully elucidated. Older post-TKA patients are often less active and show more SB due to comorbidities in the form of metabolic disorders (e.g., obesity or diabetes mellitus), cardiovascular disorders (e.g., hypertension or atherosclerosis), or nutritional deficiencies. Prolonged sedentary behaviour can lead to fat accumulation and muscle atrophy, thus increasing the risk of cardiovascular, gastrointestinal, and metabolic dysfunction diseases^36,37^. Therefore, it is necessary to reduce the comorbidity index and SB in elderly TKA patients. Healthcare professionals should advise postoperative patients to perform appropriate daily exercise to increase PA and reduce sedentary time. Patients should also be reminded to move from sitting to standing and indoor walking and to replace prolonged sedentary time spent sitting with more active activities.

The results of this study show that knee function is an influential factor in the sedentary behaviour of elderly TKA patients. This is mainly due to the fact that elderly TKA patients with low knee function have significant symptoms of joint pain and stiffness, which affect daily activities to a great extent, making patients more likely to tend to be sedentary for a long period of time to circumvent the discomfort caused by activities. At the same time, prolonged sedentary behaviour reduces the ability of the elderly to perform daily activities and take care of themselves. Previous studies^38^ reported that even at 6 months postoperatively, patients had lower bilateral extremity weight-bearing asymmetry during the transition from sitting to standing and that weight-bearing symmetry at 6 months postoperatively was associated with functional outcomes and quadriceps strength. Thus, healthcare professionals should focus on patients with high WOMAC scores when managing the diseases of elderly postoperative TKA patients, explore a scientific disease management model, and provide targeted functional exercise guidance for activities that are difficult to complete and that the patients hope to improve, so as to promote functional rehabilitation, which, in turn, will have a positive effect on the reduction of SB. Future studies could also use interactive web platforms to enable healthcare professionals to keep abreast of patients’ functional exercises^39^, and informatics-based care could provide patients with tailored health knowledge, enhance motivation and the effectiveness of patients’ home-based autonomous rehabilitation exercises through push notifications, health tracking, and rehabilitation monitoring, thereby improving the quality of rehabilitation for post-TKA patients.

Social support is considered to be helpful and protective for individuals^40^ and can come from a wide range of sources, such as friends, family, colleagues, organisations, and healthcare professionals. Social support heavily influences the initiation and maintenance of health behaviours. The results showed that older post-TKA patients with higher levels of social support tended to be more sedentary, contradicting the findings of Yasunaga et al.^41^. It was further found in the survey that elderly patients did not clearly understand the type, frequency, duration, and amount of exercise suitable for them after TKA, and that some of them had misconceptions about exercise-related knowledge, thus remaining fearful of exercise and worrying about wear and tear of the prosthetic joints or dislocation of the prosthesis due to exercise. This led them to engage in overprotective behaviours of the prosthesis, which limited their exercise behaviours and further prolonged their sedentary time. In addition, existing social and cultural expectations and attitudes towards aging in China are important barriers to reducing sedentary behaviours in elderly patients, as society generally considers “sitting” to be the primary lifestyle of the elderly. In this study, the patients were older, had multiple coexisting diseases, and were overprotected by their spouses or children, who, for safety reasons, often took over activities for them, such as grocery shopping and housekeeping, emphasising that the patients were mainly sedentary and resting, and that excessive care from family members increased patients’ SB. In view of this, healthcare professionals should strengthen the exercise-related health guidance for elderly postoperative TKA patients, while at the same time paying attention to the psychological assessment of the patients and enhancing psychological counselling for patients with a fear of exercise to assist them in establishing a positive psychological defence mechanism to reduce their fear of exercise, thus also reducing the occurrence of sedentary activities. Healthcare professionals should furthermore improve the health education and guidance for patients’ family members, informing patients that support and assistance can be given at home, but that overprotection should be avoided, and encouraging patients to engage in more physical activities that are within their ability.

Fatigue has been defined as a sense of exhaustion, lack of energy, or tiredness that is distinct from sleepiness, sadness, or weakness^26,42^. The results of this study showed that fatigue was a risk factor for increased SB in elderly postoperative TKA patients, and elderly postoperative TKA patients with a fatigue score ≥ 5 had a 4.848 times greater risk of developing sedentary behaviour than those with a fatigue score < 5. Previous studies, such as that by Hodges et al.^9^, reported the presence of fatigue in patients after TKA, with the incidence of fatigue being 43%, 33%, and 29% at 6 weeks, 6 months, and 12 months postoperatively, respectively. Few studies have focused on fatigue after TKA and the pathogenesis remains unclear. Studies have pointed out that the occurrence of postoperative fatigue may be closely linked to the levels of inflammatory factors, including interleukin-6 (IL-6), IL-10, and TNF-c, in the patient’s organism^43,44^. Changes in the levels of inflammatory factors can affect the vagus nerve, and the activation of the vagus nerve can induce a feeling of fatigue in patients, while stimulation of the vagus nerve may also be closely related to the nucleus tractus solitaries^45,46^. As patients age, their physical strength and immunity usually become weaker, making them more prone to fatigue after surgery. However, prolonged sedentary behaviour does not improve fatigue symptoms. Instead, it leads to a vicious cycle of reduced blood flow to the legs, weakened muscle strength, decreased bone density^47,48^, and sleep disturbances, resulting in increased fatigue^49^. In addition, some studies have reported that night-time fatigue is most closely related to ST, and fatigue may be an important barrier to reducing night-time SB^50^, which may be related to the long sedentary period related to patients’ screentime (watching TV, looking at mobile phones) at night. Therefore, during the clinical follow-up of elderly TKA patients, healthcare workers should also pay attention to the degree of patient fatigue and apply timely and appropriate interventions to alleviate patient fatigue, while at the same time encouraging moderate physical activities without causing or exacerbating fatigue so as to maintain patients’ physical and mental health.

### Limitations

This study is a regional cross-sectional study involving only patients discharged from a tertiary hospital in Nanjing City after joint surgery, and a self-reported SB assessment questionnaire was used, which is a simple and quick way to obtain information but may be affected by expectation bias and recall bias. It is suggested that a larger cohort study should be carried out in conjunction with electronic devices, such as accelerometers, mobile phones, or smartwatches, in the future to solve the existing study’s shortcomings. The present study did not consider the effect of family caregivers’ SB on patients, and the trajectory of SB development and changes in influencing factors in elderly patients at different stages after TKA can be investigated further in the future to make up for the shortcomings of existing studies.

## Conclusions

Sedentary behaviour is prevalent in the population of elderly post-TKA patients and should not be ignored. Comorbidities, knee function, fatigue, social support, and occupational status as risk factors for SB deserve our attention. SB is a dynamic and adjustable lifestyle. Healthcare professionals should take active measures to develop targeted intervention programmes based on the combination of relevant influencing factors, which is important for changing sedentary lifestyles and improving poor health outcomes.

However, it should be noted that in addition to the primary influencing factors mentioned above, other factors considered in this study, such as age, gender, BMI, residential status, and the presence of a sedentary lifestyle before surgery, may not show statistical significance in the multifactorial analysis due to the characteristics of the included sample, but in clinical practice, these factors still have a non-negligible effect on the SB of postoperative TKA patients.

## Data Availability

The data that support the findings of this study are available from the corresponding author upon reasonable request.
